# Intranasal DNA Vaccine for Protection against Respiratory Infectious Diseases: The Delivery Perspectives

**DOI:** 10.3390/pharmaceutics6030378

**Published:** 2014-07-10

**Authors:** Yingying Xu, Pak-Wai Yuen, Jenny Ka-Wing Lam

**Affiliations:** Department of Pharmacology & Pharmacy, Li Ka Shing Faculty of Medicine, the University of Hong Kong, Pokfulam, 21 Sassoon Road, Hong Kong, China; E-Mails: u3001464@connect.hku.hk (Y.X.); yuenpak@hku.hk (P.-W.Y.)

**Keywords:** DNA vaccine, intranasal delivery, infectious diseases, respiratory pathogens, adjuvants, mucosal

## Abstract

Intranasal delivery of DNA vaccines has become a popular research area recently. It offers some distinguished advantages over parenteral and other routes of vaccine administration. Nasal mucosa as site of vaccine administration can stimulate respiratory mucosal immunity by interacting with the nasopharyngeal-associated lymphoid tissues (NALT). Different kinds of DNA vaccines are investigated to provide protection against respiratory infectious diseases including tuberculosis, coronavirus, influenza and respiratory syncytial virus (RSV) *etc.* DNA vaccines have several attractive development potential, such as producing cross-protection towards different virus subtypes, enabling the possibility of mass manufacture in a relatively short time and a better safety profile. The biggest obstacle to DNA vaccines is low immunogenicity. One of the approaches to enhance the efficacy of DNA vaccine is to improve DNA delivery efficiency. This review provides insight on the development of intranasal DNA vaccine for respiratory infections, with special attention paid to the strategies to improve the delivery of DNA vaccines using non-viral delivery agents.

## 1. Introduction

Majority of the current licensed vaccines for the prevention of infectious diseases are live-attenuated vaccines, inactivated vaccines, or subunit vaccines. Each of them has its pros and cons. The live-attenuated vaccines can stimulate both cellular and humoral immune responses, and induce prolonged immunity that closely resembles natural infection. However, there are safety concerns associated with the use of the live attenuated virus or bacterial vaccines as they may revert to disease causing forms. It is also difficult to target multiple viral subtypes or pathogens using live-attenuated vaccines. Inactivated and subunit vaccines are safer options as they cannot replicate and do not cause disease. They confer protection mainly through humoral immune responses with little or no cellular immunity. The induced immunity lasts for a shorter period of time; therefore, supplemental doses are always required.

In recent years, DNA vaccines have attracted considerable attention as an alternative vaccination method against infectious diseases, with the potential to provide broad immune responses similar to the live-attenuated vaccines without the risk associated with the replicating micro-organisms. DNA vaccine approach relies on the *in situ* production of target antigens. Plasmid DNA encoding antigenic proteins is delivered to the appropriate tissues in the body, leading to the expression of the desired antigens, eliciting specific immunogenic responses and thereby inducing immune protection against the pathogens. Since the host cells are responsible for antigen production, the natural glycosylation and folding of the protein are warranted. Plasmid DNA encoding different bacterial and viral antigens have been tested for their immunogenicity and protective efficacy *in vivo*, confirming their clinical potential [1,2,3,4]. In addition, DNA vaccines offer several distinct advantages over conventional vaccines. The double helical structure of DNA is simple and stable at high temperature, allowing easy storage and transportation. Large-scale manufacture of DNA vaccines is convenient and relatively cheap. It only requires standard cloning of antigen coding sequence into plasmid vectors, avoiding the complex procedures of repeated culture and inactivation of infectious pathogens, or the purification of recombinant proteins. Apart from their advantageous physicochemical properties, DNA vaccines have the ability to generate the cellular immunity in addition to the humoral immunity. They are also highly flexible, encoding several types of genes including viral and bacterial antigens, as well as immunological proteins. The advantages of DNA vaccines compared to conventional vaccines are summarized in Table 1.

The field of DNA vaccination is developing rapidly. DNA vaccines are currently approved for veterinary use against various infectious diseases [5,6,7,8]. However, the results in clinical trials have been less encouraging. DNA vaccines are generally safe and well tolerated in human, but the immune response is often too low to offer sufficient protection [5,9,10,11]. In early studies, DNA vaccines alone were not able to generate T cell responses at a magnitude that was enough to protect against difficult diseases in humans [12,13]. Recent attempts still failed to overcome this problem. A plasmid pTHr DNA HIV-1 vaccine candidate evaluation in phase I clinical trials on healthy volunteers showed that it had weak immunogenicity in human. No significant HIV-1-specific cell-mediated immune response difference was found between vaccine recipients and placebo recipients, in addition to no HIV specific antibody production [14]. Another phase I trial of HIV vaccine using DNA prime-virus vector vaccine boost strategy on healthy volunteers was proved effective in eliciting T-cell responses but incapable of inducing neutralizing antibody activities [15]. In 2012, a human HIV-1 gag DNA vaccine with or without interleukin (IL)-12 and/or IL-15 plasmid cytokine adjuvant was reported to produce poor cellular immunogenicity with no vaccine-induced anti-gag humoral immune responses on healthy volunteers, which contrasted with the previous findings in macaques [11].

**Table 1:** Advantages of DNA vaccines compared to conventional vaccines.

Category	Characteristics
Design	Rapid design
	Vaccine can be developed for multiple agents in a single formulation
Production	Rapid and reproducible
	Large-scale production is relatively cheap
	Proteins are produced by host cells to ensure proper folding
Stability	Higher stability than proteins or live-attenuated microorganisms
	Ease of storage and transportation
Safety	Do not require cultivation of dangerous infectious agents
	No risk of reverting back to virulent forms
	Good safety profile in clinical trials
Immune responses	Induce both cellular and humoral immune responses similar to live-attenuated vaccines

Several strategies have been introduced to optimize DNA vaccines [16]. One of them is to enhance the DNA delivery efficiency, which is the focus of this review. DNA delivery efficiency is dependent on the administration route and the delivery system used. Mucosal surfaces are attractive sites of vaccine administration against infectious diseases as they are the portals of entry for many pathogens. Vaccination at the mucosal sites where pathogens initiate infections can be more efficacious than parenteral administration as invading pathogens may be neutralized at the front lines before generating any systemic effect. In particular, intranasal vaccine has been extensively investigated in recent years. Vaccination at nasal mucosa can stimulate respiratory mucosal immunity by interacting with the nasopharyngeal-associated lymphoid tissue (NALT) where large amounts of local lymphocytes are present. Furthermore, intranasal delivery is a needle-free, non-invasive route of administration with the possibility of self-administration. Intranasal DNA vaccination has become a promising approach in offering immune protection against various pathogens that affect the respiratory system including tuberculosis, coronavirus infection, influenza and respiratory syncytial virus (RSV). In this article, the current developments of DNA vaccine delivery systems that are specifically designed for intranasal administration against respiratory infectious diseases are discussed in detail.

## 2. Principles of DNA Vaccines

### 2.1. Mechanisms of Action

**Figure 1:**
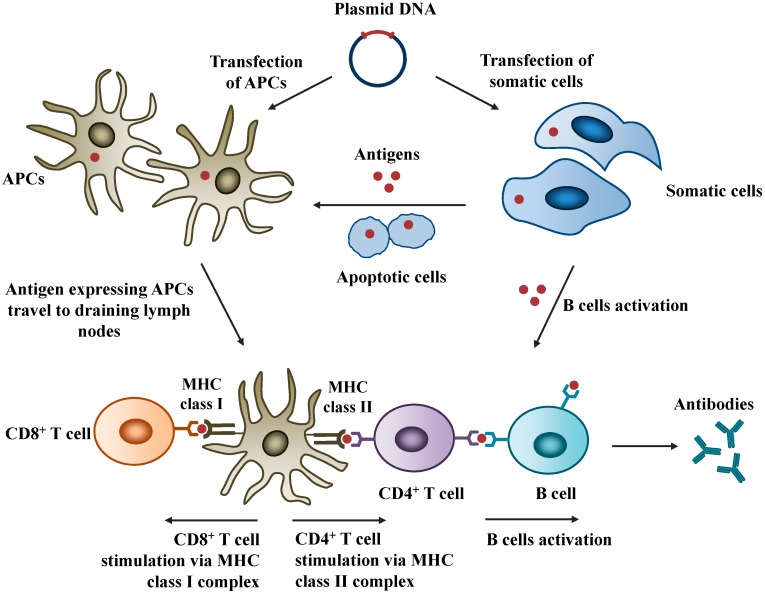
Mechanisms of action of DNA vaccines. Plasmid DNA encoding antigen of interest is transfected into antigen presenting cells (APCs) or somatic cells. Secreted antigens activate B cells, leading to antibodies production. APCs are activated by direct transfection or cross-presentation (indirect transfer of antigens). APCs then migrate to the draining lymph nodes where they present antigenic peptides to T cells via MHC-I and MHC-II.

Typically, DNA vaccines are administered by intramuscular injection although other administration routes including intradermal, subcutaneous, oral and intranasal routes are also investigated. Upon administration, somatic cells (e.g., myocytes or keratinocytes) and professional antigen presenting cells (APCs) are transfected. As the antigens are expressed intracellularly, both humoral and cell-mediated immunity can be activated to offer broad immune protection. The host-synthesized antigens become the subject of immune surveillance in the context of both major histocompatibility complexes (MHC) class I and class II molecules of APCs. Antigen expressed APCs then travel to the draining lymph nodes where they present the antigenic peptide-MHC complexes and stimulate T cells. Alternatively, B cells are activated, initiating the antibody production cascades. The major advantage of DNA vaccines is their ability to activate CD8^+^ T-cells, the cytotoxic T lymphocytes, which are important in controlling infections [17]. This action is lacking in inactivated or subunit vaccines. The induction of CD8^+^ T-cells by DNA vaccines can occur in two major ways: (i) direct DNA transfection of the APCs such as dendritic cells (DCs); (ii) cross-presentation which occurs when somatic cells such as myocytes are transfected with DNA and the expressed antigens are taken up by the APCs, or when the transfected apoptotic cells are phagocytosed by the APCs. The mechanisms of DNA vaccines are illustrated in Figure 1.

It is interesting to note that immunization may occur rapidly before a DNA vaccine expresses the antigens, and antigens expressed in somatic cells may not qualitatively be the major player in eliciting immune response. Comparing to the secondary role of somatic cells, bone marrow derived antigen presenting cell (APC) activation is an important indicator for successful induction of DNA vaccine immune response, as evidenced by the van Gogh mice experiment [18]. In this study, DNA vaccine was delivered into the skin of mouse ears by gene gun. Immune response was produced after the inoculation site (*i.e.*, the ear) was rapidly removed after immunization before antigens were expressed, indicating that mobile cells are important in elaborating immunity. In another similar study, surgical removal of the injected muscle within 10 min of DNA vaccines administration did not affect the magnitude or longevity of antibody responses to the encoded antigen [19]. Again, the results confirmed the importance of APCs over somatic cells such as myocytes and keratinocytes for eliciting immune responses.

Early studies showed that DNA vaccines are poorly immunogenic with low levels of antigen expression. To improve the immunogenicity of DNA vaccines, CpG motifs are commonly employed in the construct of the plasmid backbone [20]. Bacterial or viral DNA contains unmethylated CpG motifs, whereas in mammalian cells, CpG dinucleotide motifs are rare and are usually methylated [21]. It has been demonstrated that the unmethylated CpG motifs have immunostimulatory effect and are considered by mammalian cells as pathogen-associated molecular patterns (PAMPs). Unmethylated CpG activates innate immune cells through binding to Toll-like receptor 9 (TLR-9), which is constitutively expressed in the endosomal compartments of APCs and B cells. Once bound to the DNA that is rich in CpG motifs, TLR-9 activates the immune system by initiating pro-inflammatory response that result in the production of cytokines such as interferon (IFN)-γ and interleukin (IL)-12. However, it was found that TLR-9 deficient mice also responded to DNA vaccines, suggesting that TLR-9 may not be the sole mediator of the adjuvant effect [22,23]. DNA vaccines also interact with cytoplasmic DNA sensors including TANK-binding kinase 1 (TBK1) [24] and stimulator of IFN genes (*STING*) [25], activating TLR-independent pathways and inducing IFN-γ. These pathways are crucial in contributing to the immunogenicity of DNA vaccines.

Although persistent antigen expression of DNA vaccine is usually expected to provide long-term immune protection against infectious diseases, the effect of sustained expression of antigen must be carefully examined and controlled. It has been reported that prolonged expression of antigen may lead to the switch of type-1 IFN from an antiviral cytokine to an immunosuppressive cytokine [26], or may deplete the pool of memory T cells [27].

To evaluate the efficacy of DNA vaccine in humans, serum antibody titer or the enzyme-linked immunoSpot (ELISpot) assays are the commonly employed methods to measure the immunogenic response, although the induction of antigen-specific immune effectors by an immunization process does not imply that these antibodies or cytokines represent surrogates or correlates of vaccine efficacy. In early stage of vaccine development, *in vitro* serum antibodies and ELISpot assays are the direct detectable indicators of the clinical potential of a vaccine formulation. At later stage of development, morbidity and mortality (especially the improvement of survival rate after vaccination) in animals upon target pathogen challenge is a more certain way to confirm the protective efficacy of vaccines [28,29], as the ultimate goal of vaccine is to prevent the targeted disease. The efficacy of vaccine such as influenza vaccine could be monitored in human during subsequent influenza epidemic season [30,31] or challenged with a controlled influenza virus [32]. However, some lethal virus challenge studies are difficult to conduct directly on human. Hence, the measurement of antibodies production and immune responses in humans remain the most direct way to assess vaccine efficacy. Longer study is required to investigate if the vaccine is indeed able to prevent disease.

### 2.2. Safety of DNA Vaccines

Safety is always a primary concern with any vaccine products. DNA vaccines are generally considered to be safer than conventional vaccine approaches as they lack the risk of reversion to a disease causing state or secondary infection. Similar to other gene therapy, the major safety issue related to DNA vaccines is the risk of integration of the plasmid DNA into the host genome, causing insertional mutagenesis, which may lead to the inactivation of tumour suppressor genes or activation of oncogenes, resulting in devastating adverse effects. According to preclinical and clinical studies, there is little evidence of genomic integration following DNA vaccines administration, and the risk of integration is found to be significantly lower than the spontaneous mutation rate [33,34,35,36].

Another safety issue of DNA vaccines is related to the development of anti-DNA immune responses. Animal studies showed that there is no increase in anti-nuclear or anti-DNA antibodies after DNA vaccination. In clinical trial studies, signs and symptoms of autoimmunity, and the markers of autoimmunity are sometimes monitored in the human subjects. There has been no evidence that autoimmunity is associated with DNA vaccines [5,37,38,39]. It has been suggested that proper purification of *E. coli* can effectively prevent pathogenic anti-DNA antibody production [2,40]. Antibiotic resistance is another issue related to DNA vaccines. Typically, large-scale manufacture of plasmid DNA involves the use of antibiotic-resistant marker. There is a safety concern that resistance to the same antibiotic might be introduced. In response to this issue, antibiotic-resistance genes in DNA vaccine should be restricted to those that are not used to treat human infections. Alternatively, the use of antibiotic-resistance genes should be avoided completely [41]. Another concern of DNA vaccines is the development of tolerance to the encoded antigen, which appears to be age-related. Newborns have immature immune system and are more likely to develop tolerance rather than protection when exposed to foreign antigens. In contrast, immunity instead of tolerance occurred when DNA vaccines are administered to young animals [42,43,44].

In recent years, there has been an increasing concern that vaccination in general may induce harmful systemic inflammation, which may lead to increase of cardiovascular risk [45,46,47,48,49]. DNA vaccine is still considered as a relatively new approach to vaccination but its potential to induce systemic inflammation must not be overlooked. It was reported that little or no local inflammatory infiltration was observed at the DNA vaccine injection site, especially after the acute effects of the vaccination have disappeared [9]. The first clinical trial of a DNA-based vaccine for HIV-1 infection was published in 1998 in 15 asymptomatic HIV-infected patients who were not using antiviral drugs. The immunization was well tolerated with neither local, systemic reaction nor laboratory abnormalities were detected after three doses of vaccines [38]. In addition, no patient developed anti-DNA antibody or muscle enzyme elevations. No consistent change of CD4^+^ or CD8^+^ lymphocyte counts occurred. Another early experiment conducted on pigs showed that electroporation of DNA vaccines was more efficient in enhancing immune response, but also stimulated inflammatory response and accompanying cellular infiltration, whereas the conventional intramuscular injection of DNA vaccines only showed low gene expression and low inflammatory cell infiltration [50]. It was suggested that improved antigen presentation was one of the possible mechanisms by which increased inflammatory cell infiltration may enhance immune responses to DNA vaccines delivered with electroporation. However, the long-term safety effect was not investigated.

Overall, many recent preclinical studies and clinical trials have indicated that DNA vaccines are generally well tolerated with good safety profile, and no systemic inflammation was reported [11,14,51,52,53,54]. Nonetheless, DNA vaccines are relatively new vaccination approaches and yet to be approved in human use, the long term safety of their uses must be thoroughly evaluated for routine prophylactic and therapeutic use in human, especially when new delivery systems or adjuvants are introduced into the formulation.

## 3. Intranasal Vaccines

Conventional vaccines are usually administered by parenteral injections which mainly target the systemic immune system, eliciting only weak mucosal immune response. When the vaccines are delivered directly to the mucosal site, mucosal immune response can be more efficiently potentiated. In particular, nasal mucosa has attracted considerable attention as the site of vaccination in recent years, including DNA vaccines, due to several distinct advantages. However, there are also some formidable barriers that need to be overcome to allow successful development of intranasal DNA vaccines.

### 3.1. Intranasal Route of Administration

The intranasal route of drug administration has been frequently used to treat local conditions such as nasal congestion and allergy. Intranasal administration is characterized by easy administration, rapid onset of action and avoidance of first-pass metabolism. The needle-free administration route is non-invasive and can avoid the risk of spreading blood-borne infections, which is a particular problem in developing countries. These desirable features lead to the exploration of the systemic delivery of polar drugs or biomolecules including vaccines that are not feasible in other administration routes.

Intranasal vaccination has been investigated for over a decade. The majority of currently available vaccines are administered by intramuscular, subcutaneous or intradermal injection. Although these parenteral routes of administration are effective in inducing systemic immune responses, they are ineffective in inducing local immunity at mucosal sites. As many as 70% of pathogens infect human through the mucosal surfaces [55]. Mucosal vaccination could provide better protection than injectable vaccines against infectious diseases by inducing both systemic and mucosal immunity [56,57]. Since the strongest immune response is usually induced at the vaccination site and the adjacent mucosal sites, intranasal immunization is able to elicit protective immune response effectively in the lungs and the upper respiratory tract [58]. Nasal mucosa appears to be an appropriate site of vaccine administration against respiratory infectious diseases, not only because the nasal cavity is the first site of contact with inhaled macromolecules and a common site of infection by respiratory pathogens, it can also stimulate respiratory mucosal immunity by interacting with the NALT. Current, licensed intranasal vaccines include FluMist^®^, a live-attenuated vaccine that targets influenza types A and B [59]; and NASOVAC^®^, a live-attenuated vaccine that targets H1N1 influenza virus [60]. Apart from live-attenuated vaccine, intranasal route of administration is also favorable to protein-based vaccination, as evidenced by many studies including the intranasal pneumococcal protein immunization against pneumonia [61], and a recent study on the intranasal respiratory syncytial virus (RSV) vaccine based on a recombinant fusion protein [62]. With the success of intranasal live-attenuated virus vaccine and the promising effect of protein-based vaccine, it is highly plausible that DNA vaccines can adopt the same delivery route to achieve efficient immunization.

### 3.2. Mechanisms of Nasal Mucosal Immune Protection

**Figure 2:**
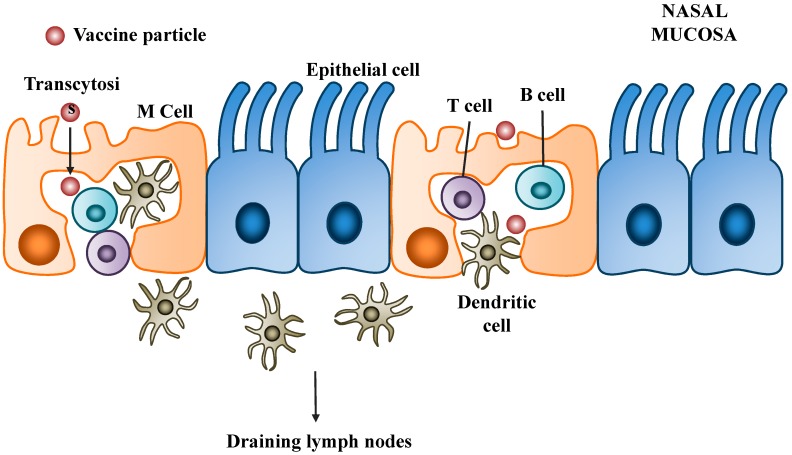
The initiation of mucosal immune response. Particles are taken up by M cells and transported to the underlying immune cells through transcytosis. Dendritic cells (DCs) at mucosal site may migrate to the nearest draining lymph node to present antigen to T cells.

It is well established that mucosal vaccination can induce humoral and cell-mediated immune response systemically as well as at mucosal sites [56,57]. Immune response induced by mucosal vaccination is mainly initiated at specific mucosa-associated lymphoid tissue (MALT). The MALT lining the nasal cavity is known as the nasopharyngeal-associated lymphoid tissue (NALT), which include the Waldeyer’s ring of tonsils, adenoids and a collection of isolated subepithelial lymphoid follicles [63]. The NALT is rich in immunocompetent cells, including B cells, T cells and phagocytic APCs such as macrophages and DCs [64]. In addition, the overlying epithelium of mucosal follicles forms a specialized cell layer. These cells have microfolds on their apical surface and are known as microfold cells (M cells). M cells play a crucial role in the initial phase of induction of mucosal immune responses. Therefore, M cell targeting is an important strategy to achieve mucosal immunity [65,66]. M cells are efficient in taking up particles from the epithelial surface, transporting them across the cells and releasing them to the underlying extracellular space. This process is known as transcytosis. At their basal surface, the cell membrane of M cells is extensively folded around the underlying immune cells including B cells, T cells and APCs, which take up the particles released from M cells and process them for antigen presentation. The initiation of mucosal immune response is summarized in Figure 2.

Upon B cells activation following nasal vaccination, the production of antigen-specific secretory immunoglobulin A (sIgA) is triggered. sIgA is a critical component in the mucosal immune system. It is protease resistant, and can effectively bind and neutralize pathogens and their toxic products on the nasal mucosa surface despite the protease rich environment, thereby preventing the pathogens from breaching the mucosal barrier [67]. Local immunoglobulin G (IgG) production is also detected after mucosal vaccination [68] and partly contributes to the neutralization of pathogens. However, IgG concentration is around 30–100-fold lower than that of the sIgA due to its susceptibility to protease degradation [56]. Indeed, sIgA provides the first barrier to pathogens invasion, so induction of potent sIgA response is an important goal of mucosal vaccination. In addition, nasal immunization can also result in the production of serum IgA and serum IgG, which can potentially neutralize pathogens that enter the mucosa and prevent systemic spread. When the DCs at the mucosa are presented with antigens, the activated cells may migrate to the proximal draining lymph node and disseminate immune responses to other sites of the body. Apart from the humoral immune response, cell-mediated immune response is also induced after mucosal vaccination. Although the cytotoxic T cells in the mucosal tissues may not prevent pathogen entry, they are crucial for the clearance of pathogens [56].

Overall, cells of NALT are involved in the regulation of both humoral and cell-mediated immune responses locally and also systemically, offering a broad immune response. Since the nasal mucosa is an important portal of entry for respiratory pathogens, the nasal route has become attractive for the administration of vaccines by reinforcing the nasal mucosal immune response.

### 3.3. Barriers to Intranasal DNA Vaccines

The defense mechanism of the nasal cavity presents a significant barrier to the entry of pathogens and potentially harmful substances; however, it has also become an important barrier to intranasal DNA vaccines. The nasal mucosa, which constitutes the outmost layer of the nasal passage, consists mainly of ciliated columnar cells, non-ciliated columnar cells, goblet cells and basal cells. The proportions of these cells vary in different regions of the nasal cavity. Nasal mucus, which is produced by goblet cells and submucosal glands, provides a protective physical barrier to foreign materials. It is a highly viscous, gel-like heterogeneous mixture that contains glycoproteins, enzymes, immunoglobulins, salts, proteins and lipidic components [69]. DNA vaccines that are administered to the nasal cavity have a propensity to be trapped by the nasal mucus, leading to enzymatic degradation. The effect of mucus depends on its viscosity and pore size, which affect the diffusivity of agents delivered to mucosal surfaces. In addition, the entrapped DNA vaccines may also be removed by the mucociliary action of the ciliated cells that drives the overlying mucus layer continuously towards the nasopharynx, clearing the mucus from the nasal passage, resulting in short residence time at the mucosal surface. Another challenge of intranasal vaccine is that the vaccine formulation may be diluted in mucosal fluids, and bulk fluid may limit effective deposition onto the epithelium of the mucosal system.

To overcome these barriers, a safe and efficient DNA delivery system must be developed. Ideally such delivery system should target the mucosal APCs for antigens processing that lead to specific B and T cell activation. The ultimate goals of DNA delivery systems are to facilitate the uptake of DNA to the target tissues and cells, protect DNA from enzymatic degradation, increase the residence time of the formulation in the nasal cavity, enhance the expression of the antigens and to increase the immune response without compromising safety. The different DNA vaccine delivery systems currently being investigated for intranasal administration are discussed in detail in Section 5.

### 3.4. Potential Risks of Intranasal Vaccine to the Central Nervous System

Despite the numerous merits of intranasal immunization, the potential hazard of nasal vaccines must not be overlooked. The concern of the safety of intranasal vaccination was raised after an intranasal inactivated influenza vaccine called NasalFlu, developed by Berna Biotech, was found to be associated with Bell’s palsy, a temporary neurological paralysis of one side of the face [70]. NasalFlu consists of influenza virosomes which are formulated to contain hemagglutinin (HA) and neuraminidase (NA) antigens, as well as heat-labile enterotoxin (LT) from *E. coli* as mucosal adjuvant. Since parenteral administration of inactivated influenza vaccine did not confer an increased risk of Bell’s palsy, nor the natural influenza virus infection, it was soon concluded that adjuvant LT from *E. coli.* was the culprit of these cases. For this reason, FluMist^®^ and NASOVAC^®^—both are intranasal live-attenuated influenza vaccines without enterotoxin as adjuvants—do not appear to confer an increased risk for this condition.

Understanding the pathogenesis of the Bell’s palsy that was connected to NasalFlu has become an important research focus for vaccine development. Following intranasal administration in mice, enterotoxins were found in the olfactory nerve and the olfactory bulb for an extended period. Since the olfactory epithelium is the only part of the central nervous system (CNS) that is exposed to the external environment, drugs and nanoparticles, including intranasal vaccines, may bypass the blood brain barrier and enter the CNS through olfactory transmission. There is a reason to concern the neurotoxic effects of vaccine containing enterotoxin adjuvant for intranasal administration. While the nasal delivery of neuronal-binding LT-derived adjuvants is inadvisable [71], other toxin-derived adjuvants such as cholera toxin-derived CTA-DD and double mutant cholera enterotoxin (CT) are claimed to be safe and effective adjuvant candidates without causing any inflammation or CNS toxicity [72,73]. Nevertheless, thorough evaluation must be performed with the use of toxin derivatives as intranasal vaccine adjuvants. Tremendous efforts are now focusing on the development of alternative adjuvants with better safety profile.

## 4. Clinical Applications of DNA Vaccines against Respiratory Infections

Mankind has been haunted by respiratory infectious diseases for aeons. They have created public health concerns since ancient times. With the emergence of new or drug-resistant strains, it is becoming a challenge to protect the public from infections using conventional vaccine methods. DNA vaccines have huge potential for the prevention of respiratory infections due to their ability to offer broad immunity, the relatively rapid process of designing new DNA vaccine construct and the possibility of large-scale production in a short period of time. In this section, four pathogens that cause severe diseases in the airways are highlighted, including tuberculosis, coronavirus, influenza and respiratory syncytial virus, with a brief discussion of current DNA vaccine development against these infections.

### 4.1. Tuberculosis (TB)

Tuberculosis (TB) is a bacterial infectious disease caused by *Mycobacterium tuberculosis* which is transmitted by respiratory aerosols. TB has become a major public health problem that threatens the progress made in TB care and control in the world. The only TB vaccine currently available is an attenuated strain of *Mycobacterium bovis*, Bacillus Calmette-Guérin (BCG), developed in the 1920s. However, its efficacy against adult pulmonary TB remains controversial [74]. With the emergence of drug-resistant TB and increasing rates of HIV/AIDS and TB co-infection, a new effective TB vaccine is urgently in need. Effective protective immunity to *Mycobacterium tuberculosis* requires cell-mediated immune responses, including both CD4^+^ and CD8^+^ T cells [75,76,77]. Since DNA vaccines have the ability to induce strong cellular immunity, it has become an attractive vaccine approach against TB. The first two studies that reported promising protective effect with DNA vaccine against tuberculosis were conducted in mice using plasmid DNA encoding antigen 85A (Ag85A) of *Mycobacterium tuberculosis* [78] and the 65 kDa heat-shock protein of *Mycobacterium leprae* (hsp65) [79]. Other different antigens such as Ag85B ESAT-6, IL-23, MPT64, PstS-3 and other fusion proteins have also been explored as DNA vaccines against tuberculosis [80,81,82,83,84,85]. Most of these DNA vaccines encode mycobacterial proteins that are either secreted in mycobacterial culture filtrate or exposed on the mycobacterial cell-wall surface.

### 4.2. Coronaviruses (CoV)

Coronaviruses (CoV) are potentially lethal pathogens, characterized by the presence of spike proteins on the viral surface. Two new strains, severe acute respiratory syndrome CoV (SARS-CoV) and Middle East respiratory syndrome CoV (MERS-CoV), have been identified. Both of them could cause acute respiratory distress syndrome (ARDS) and are associated with high mortality rates [86]. CoV vaccines have historically exhibited poor capacity for cross-protection [87], the development of safe, broad spectrum and effective vaccines that can be rapidly made available during an emerging epidemic is required. Currently, there are no approved vaccines for human CoV infections, and most of the studies have focused on the SARS-CoV. Spike and nucleocapsid proteins, which are the immunodominant CoV proteins, are the antigens of interest for vaccine development [88,89]. DNA vaccines that encode nucleocapsid protein induced strong cell-mediated immunity but are not protective after high titer of viral challenge [90,91]. In addition, nucleocapsid DNA vaccine could induce delayed-type hypersensitivity even in the absence of an antibody response. This effect was not observed with spike protein DNA vaccines.

### 4.3. Influenza

Influenza is caused by orthomyxoviruses which are RNA viruses that affect mainly the upper respiratory tract. In recent years, zoonotic or variant influenza has become a serious threat to human health, including the avian influenza virus H5N1 and H9N2, and the swine influenza virus H1N1 and H3N2. Although these animal viruses are distinct from human influenza viruses and do not usually transmit between humans, they may still occasionally infect humans and cause severe pneumonia and even death. Furthermore, if such a virus acquired the capacity to spread easily among people, it could start an epidemic or even a pandemic [92,93]. To protect the populations from influenza infection, highly effective, broad-spectrum influenza vaccines that could be prepared rapidly are in demand. Current influenza vaccines mainly target the induction of antibodies against the viral glycoproteins, particularly surface glycoproteins hemagglutinin (HA) and neuraminidase (NA). Antibodies to HA neutralize the infectivity of the virus while antibodies to NA prevent the release of the virus from the infected cells. Apart from surface glycoproteins, internal proteins such as nucleoprotein (NP) and matrix protein (M1), as well as the ion channel protein (M2), which are highly conserved between and within different subtypes, have also become very attractive target antigens for vaccines to provide broad, cross-strain protection [94,95].

DNA vaccines can potentially solve the mismatch problem by shortening the lag time [96], which is particularly useful when facing influenza pandemic. In addition, the strategy of combined immunization with DNA vaccines encoding surface protein (e.g., HA) and internal protein (NP and M1) could offer better protection against influenza virus than single DNA vaccine alone in mice and ferrets [97,98,99,100]. With the success of DNA vaccines in various animal models, several phase I & II clinical trials on DNA vaccine against influenza have been being carried out. Results so far have been encouraging, demonstrating both safety and immunogenic response in human [32,52,101].

### 4.4. Respiratory Syncytial Virus (RSV)

Respiratory syncytial virus (RSV) is a single stranded RNA pneumovirus which belongs to *Paramyxoviridae* family. It accounts for one of the leading pathogeneses of lower respiratory tract infections and hospitalization in infants and young children [102], as well as the elderly and high-risk population [103]. Immunity against RSV is dependent on the induction of antibody responses. In addition, CD8^+^ T cells responses have been shown to reduce disease severity [104]. Although maternal antibodies appear to protect infants against infection, their amount gradually decreases within the first few months of life. Human RSV lacks an approved vaccine or an antiviral therapy. To prevent infant and childhood infection, vaccine should be able to induce immune responses rapidly after birth. This could be a challenging task because the immune system at the first few months of life is immature, and the persistence of maternal antibodies may limit the induction of infant antibodies responses. Three RSV proteins, namely the fusion (F) protein, attachment glycoprotein (G) and matrix protein (M2), are the leading candidates for RSV vaccine development [105,106,107,108,109,110,111].

## 5. DNA Vaccine Delivery System for Intranasal Administration

Delivery is one of the major barriers to DNA vaccine. Administration of naked DNA is usually inefficient with only a small fraction of DNA being taken up by the cells and subsequently expressed [112]. This is because DNA is a negatively charged, hydrophilic macromolecule; it is incapable of crossing the biological membrane unassisted. Therefore, a safe and efficient DNA delivery system is sometimes employed as adjuvant to facilitate efficient cellular uptake of DNA vaccines, promote DNA release inside the cells, induce high level of antigen expression and hence immune responses. Physical method such as gene gun, also known as the particle-mediated epidermal delivery, has been studied to deliver DNA to the skin [101,113,114,115]. Gold beads coated with DNA vaccines are discharged directly into the cytoplasm and nuclei of skin cells. This method of delivery has enjoyed some success, but is not applicable for intranasal administration. Considerable efforts have been made to improve the efficacy by developing effective DNA delivery systems for intranasal vaccines. Formulation of DNA vaccines in synthetic non-viral vectors such as polymeric nano-/micro-particles and liposomes has been reported to increase the uptake of plasmid DNA by cells, increasing immunogenicity in animal models and humans. Additional adjuvants may also be used to further improve the immunogenicity of these delivery systems.

### 5.1. Cell-Specific Targeting

The elicitation of immune responses of DNA vaccines mainly relies on professional APCs that present antigens to both B cells and T cells. To ensure good immune response, the DNA vaccine delivery systems should be able to target APCs. In addition, M cells in NALT, which is a major site of pathogen entry, are also a target of DNA vaccine.

APCs are a heterogeneous group of immunocompetent cells that mediate immune response by processing and presenting antigens to the T cells. T cells recognize only antigenic peptide fragments on the surface of APCs through the T cell receptors. Helper T cells recognize antigen in association with class II MHC proteins, whereas cytotoxic T cells recognize antigen in association with class I MHC proteins. An additional co-stimulatory signal is then produced by the APCs, leading to the activation of T cells. Non-professional APCs lack the co-stimulatory signaling, and therefore do not simulate T cells sufficiently. There are three types of professional APCs, namely DCs, macrophages and B cells. Among them, DCs have the broadest range of antigen presentation and are considered as the most efficient cells for induction and regulation of immune responses. They play a central role in bridging the innate immune system with the adaptive immune system [116,117,118]. To achieve efficient DNA vaccination, it is logical to target the plasmid DNA to DCs where the encoded antigen could be expressed endogenously.

DCs express a large number of surface receptors such as C-type lectin receptors (CLRs) and TLRs, which are engaged in the recognition of pathogens. It has been reported that targeting antigens to receptors on DCs can significantly enhance immune responses [119]. These receptors could be exploited for DNA vaccine targeting with the aid of antibodies or natural ligands. In particular, CLRs are endocytic receptors which recognize carbohydrate structures that resemble pathogen cell-wall components. They are responsible for internalizing pathogens. One of the most commonly studied receptors for vaccine targeting is the DC C-type lectin receptor 205 (DEC-205) which is specifically expressed on DCs. However, ligands for DEC-205 have yet to be identified. Several studies have demonstrated the employment of anti-DEC-205 antibodies to achieve DC targeting for DNA vaccines, including intranasal immunization [120,121,122]. Another identified DC-specific target is C-type lectin domain family 9 member A (CLEC9A). Activation of CLEC9A leads to the stimulation of antibody production [123]. Alternatively, DCs could be targeted by using the natural ligands to the mannose receptors [124]. However, the effectiveness of these targeting components in mucosal vaccines remains to be investigated.

There are also several molecules being investigated to target APCs in general. One of the most widely studied molecules is the Flt3 ligand. Flt3 ligand is a growth factor that stimulates the proliferation of hematopoietic cells. It binds to the fms-like tyrosine kinase receptor Flt3. Flt3 expression is, in hematopoietic tissue, restricted to CD34^+^ progenitors, including DC progenitors. *In vivo* treatment of Flt3 ligand is found to up-regulate the number of DCs, but not their activation [125,126]. Furthermore, Flt3 ligand treatment could also enhance immune response when delivered via the mucosal route [127]. It has been reported that when plasmid DNA encoding Flt3 ligand was co-administered with plasmids encoding protein antigens, effective immune responses were induced [128]. In addition to its APCs targeting ability, Flt3 ligand is an efficient and safe mucosal adjuvant that facilitated expansion of DCs following nasal administration [129,130].

As discussed in Section 3.2, improving M cells uptake is another strategy to enhance vaccine immunity. Effective mucosal immunity often correlates with the uptake of antigen by mucosal inductive tissues, such as NALT in the upper respiratory tract following intranasal immunization. Since M cells are responsible for antigen sampling on the mucosal surface for eventual antigen presentation to mucosal B and T cells, targeting of vaccines to M cells can be an effective method to achieve strong immune response. Particle size is an important parameter for M cell uptake. A number of studies have been conducted to identify the optimal particle size for cellular uptake of the mucosal system. Some studies suggest that particle size of less than 1 μm is optimal for oral vaccine delivery for Peyer’s patch M cell uptake [131,132,133]. Another NALT nanoparticle uptake study also suggested that particles with sub-micron size are optimal for mucosal M cells uptake [134]. It is generally accepted that NALT M cells can uptake nanosized particles rapidly with no definite size range being established [135]. Apart from cellular uptake, particle size also affects the kinetics of lymphatic drainage. It appears that nanoparticles less than 200 nm are more readily transported by the draining lymph compared to larger particles [136].

Apart from controlling particle size to achieve specific targeting passively, inclusion of targeting ligand could also increase uptake by M cells [133,137]. A number of pathogens including reovirus [138], *Salmonella typhimurium* [139] and *Mycobacterium tuberculosis* [140] target M cells as a mode of entry into the host. By identifying the key molecules expressed by these pathogens that are crucial for their invasion, it would be extremely helpful to design an effective delivery system for M cells targeting [141]. One example is related to reoviruses which target M cells using their surface protein sigma-1 (σ1). In this regard, Wu *et al.* reported an M cell targeting DNA vaccine delivery system consisting plasmid DNA and the recombinant protein σ1 as targeting ligand which was covalently attached to poly-l-lysine (PLL) for intranasal vaccination in mice. The results showed significant mucosal sIgA production as well as enhanced cell-mediated immunity [65]. Other ligands such as Co-1 peptide [142], Claudin 4 targeting peptide [134,143] and M cell specific monoclonal antibody (NKM 16-2-4) [144] have been investigated for M cell targeting in mucosal protein vaccine, with the potential to be explored for mucosal DNA vaccine delivery. However, mucosally induced tolerance may develop with M cell targeting delivery system. Following nasal administration of protein σ1 genetically conjugated with ovalbumin, systemic unresponsiveness was induced instead of mucosal IgA immunity [145]. Therefore, special attention must be paid with the development of M cell targeting delivery system.

### 5.2. Polymers

High versatility is one of the attractive features of polymer-based DNA delivery systems. Cationic polymers can form complexes (polyplexes) with nucleic acids through electrostatic interaction. Polymer synthesis is relatively cheap and is easy to scale-up. Particle size and surface properties of polymeric particles can be controlled by using different polymers and fabrication methods in order to optimize their cellular uptake and transfection efficiency. The polymeric particles can also be modified to include specific function groups or ligands to enhance immune responses.

#### 5.2.1. Polyethylenimine (PEI)

**Figure 3:**
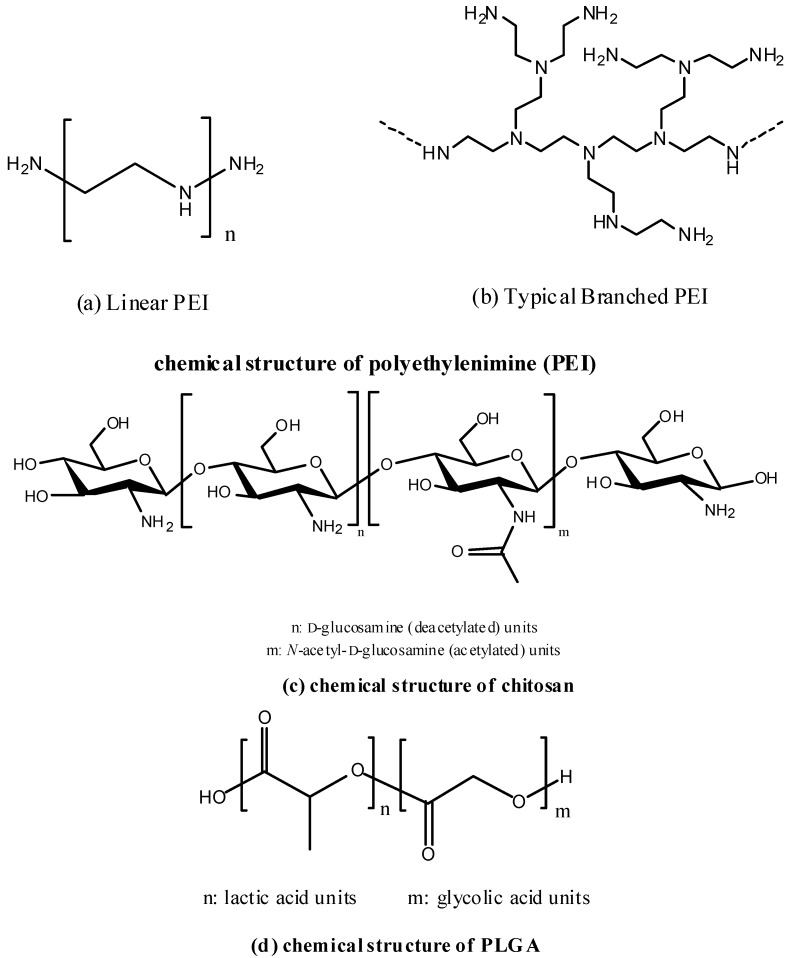
Chemical structures of Polyethylenimine (PEI) (**a**,**b**), Chitosan (**c**) and Poly(lactic-co-glycolic acid) (PLGA) (**d**).

Polyethylenimine (PEI) (Figure 3) is one of the early generation polymers being investigated for gene delivery. It has high transfection efficiency and is frequently regarded as the gold-standard of non-viral gene delivery vectors. PEI has high pH buffering capacity, which allows its cargo to escape from endosomal entrapment via a mechanism known as “proton sponge hypothesis” [146]. Transfection efficiency of PEI depends on its molecular weight and the level of branching. Shim *et al.* described the use of a simple method to prepare PEI (25 kDa)–DNA complexes for vaccine delivery [147]. Plasmid DNA encoding SARS-CoV spike protein without transmembrane domain was employed in the study. Mice that were immunized intranasally with the PEI–DNA vaccines produced significantly higher systemic spike protein specific IgG and mucosal secretory IgA in the lung compared to those immunized with naked DNA. Furthermore, cellular immune responses were detected with an improvement of specific T cell responses. In another study, Torrieri-Dramard *et al.* demonstrated the utilization of PEI (*in vivo*-jetPEI^®^) as DNA vaccine carrier for intranasal administration [148]. Plasmid DNA encoding HA from influenza A viruses was used. The intranasal administration of the PEI/DNA vaccines induced cellular and humoral immune response capable of providing protective immunity against a divergent virus of H5N1 subtype in mice. The protection could be further improved by including the plasmid DNA encoding NA.

Although PEI appeared as a promising vector for gene delivery including DNA vaccines, the cationic PEI is highly charged and non-biodegradable, and it often encounters toxicity problems which cannot be ignored. In this regard, many groups are developing low toxic or biodegradable PEI derivatives for gene delivery application. Mann *et al.* has developed a PEI derivative, deacylated PEI (dPEI), as DNA vaccine delivery agent [149]. dPEI is a nearly fully hydrolysed linear PEI with 11% additional free protonatable nitrogen atoms, enabling more efficient binding with DNA, reduced toxicity and high transfection efficiency. It is an effective DNA vaccine carrier for pulmonary delivery to elicit both systemic and mucosal immune responses, and offers protection against influenza challenge in vaccinated mice. This system has a potential to be exploited for intranasal administration.

#### 5.2.2. Chitosan

Chitosan (Figure 3) is a natural polysaccharide that has been frequently studied for drug delivery. It is derived from chitin which is found abundant in crustacean. It is biodegradable and biocompatible with low toxicity [150,151]. The properties of chitosan can be tuned by changing its molecular weight and degree of deacetylation. Because of its cationic nature, chitosan has strong binding affinity with nucleic acids, making it a suitable candidate for DNA delivery agent. In addition, chitosan and its derivatives are found to display strong mucoadhesive property, making them particularly suitable to facilitate intranasal delivery [152,153,154]. Moreover, chitosan was also reported to have an immunostimulating effect, such as increasing the accumulation and activation of macrophages, promoting resistance to infections by cytokines, and enhancing cytotoxic T cell response [155,156].

A number of studies have described the use of chitosan nanoparticles to deliver DNA vaccine formulation for intranasal administration. Kumar *et al.* reported the exploitation of chitosan nanoparticles to deliver DNA vaccine against acute respiratory syncytial virus (RSV) infection [156]. A cocktail of plasmid DNA encoding a number of RSV antigens was used to complex with chitosan to form nanoparticles. Following the nasal vaccination in mice, high levels of serum IgG and mucosal IgA antibodies, as well as cytotoxic T cells responses were induced. There was also an elevated lung-specific production of IFN-γ with antiviral action. A single dose of DNA vaccine was able to decrease the RSV titers by 100-fold on primary infection. Similar study was performed by another group who described the use of chitosan nanoparticles to deliver plasmid DNA encoding a T cell epitope from the M2 protein of RSV [157]. It was found that intranasal administration of the formulation in mice induced specific cytotoxic T cell response that was comparable to those induced via intradermal immunization. Following RSV challenge of the nasal immunized mice, the virus load in lungs was significantly reduced.

In a recent study, Raghwanshi *et al.* investigated a sophisticated DC targeted chitosan nanoparticle system for nasal DNA immunization against SARS-CoV [122]. The chitosan nanoparticles were surface functionalized with ligands to achieve DC selective targeted delivery. DEC-205 receptor is C-type lectin receptor found in DCs for recognition and uptake of pathogens. The authors developed a bifunctional fusion protein (bfFp) vector which consists of truncated core-streptavidin fused with anti-DEC-205 single chain antibody. The core-streptavidin arm of the fusion protein binds with biotinylated chitosan nanoparticles while anti-DEC-205 scFv imparts targeting specificity to DC DEC205 receptor. Plasmid DNA encoding nucleocapsid protein of SARS-coV was loaded into the chitosan nanoparticles. Following intranasal administration of the DC targeted nanoparticles in mice, the levels of mucosal IgA and systemic IgG against nucleocapsid proteins were significantly enhanced, whereas no mucosal and systemic immune responses were detected when the naked plasmid DNA was intranasally administered. The results showed that such DC targeting delivery system could be a promising strategy for low-dose nasal DNA vaccines.

To enhance the transfection efficiency of chitosan for intranasal administration, thiolated chitosan derivative has been introduced. Thiolated chitosan derivative has strong mucoadhesive properties due to the formation of disulphide bonds between thiol groups of the modified polymer and cysteine-rich subdomains of glycoproteins in the mucus layer, leading to an improvement in mucoadhesion of up to 140-fold when compared to unmodified chitosan [158]. Improved and sustained gene expression could be achieved both in vitro and in vivo with thiolated chitosan derivative [159]. This technology has a huge potential to be adopted for intranasal DNA vaccine delivery.

#### 5.2.3. Poly(lactic-co-glycolic acid) (PLGA)

Poly(lactic-co-glycolic acid) (PLGA) (Figure 3) is a synthetic biodegradable copolymer that has been extensively investigated for the delivery of different therapeutic agents including proteins and nucleic acids [160]. Due to its biocompatibility and excellent safety profile, PLGA is approved by the FDA in various drug delivery systems for human use. Since the degradation rate of PLGA can be adjusted by modifying the molecular weight of the polymer and the lactic acid to glycolic acid ratio, the rate of drug release can also be controlled accordingly. However, the negative charge and hydrophobic nature of PLGA limit its interaction with the negatively charged DNA. Cationic surface modification of PLGA micro/nanoparticles using polycations such as PEI and chitosan can overcome this problem and allow efficient nucleic acids delivery [161,162,163,164]. This strategy has also been applied to the delivery of DNA vaccines. Oster *et al.* first employed the use of micro-particles consisting PLGA and PEI as DNA vaccine carrier for injection [165]. Later on, Wang *et al.* reported the use of chitosan coated PLGA nanoparticles to deliver plasmid DNA encoding FMDV (foot-and-mouth disease virus) capsid protein together with IL-6 as mucosal adjuvant for intranasal vaccination [166]. Chitosan coated PLGA nanoparticles were first prepared by emulsion-diffusion-evaporation technique [167], followed by the incorporation of plasmid DNA to the nanoparticles by simple complexing. The samples were then freeze-dried with mannitol before use. After intranasal administration in guinea pigs and rats, both cellular and humoral immune responses were detected. IL-6 was also found to be an effective mucosal adjuvant which significantly enhanced mucosal and systemic immune responses. More importantly, the vaccines could offer some immune protection to animals against FMDV challenge.

### 5.3. Liposomes

Liposomes are vesicles comprised of phospholipid bilayers. They have been extensively investigated for delivering DNA into mammalian cells as well as vaccine adjuvants. The duo functions make them an excellent carrier system for DNA vaccine [168,169,170,171]. For DNA delivery, the negatively charged plasmid DNA can be absorbed to the surface of cationic liposomes through electrostatic interaction to form complexes. Alternatively, DNA can be encapsulated in the aqueous core of the cationic, non-ionic or anionic liposomes. In general, transfection efficiency of cationic liposomes is superior to their non-ionic or anionic counterparts [172], whereas anionic liposomes provided enhanced antibody responses [171]. Surfaces of the liposomes can be decorated with targeting ligands or antigenic components to improve the immune response in vaccine formulation [173].

**Figure 4:**
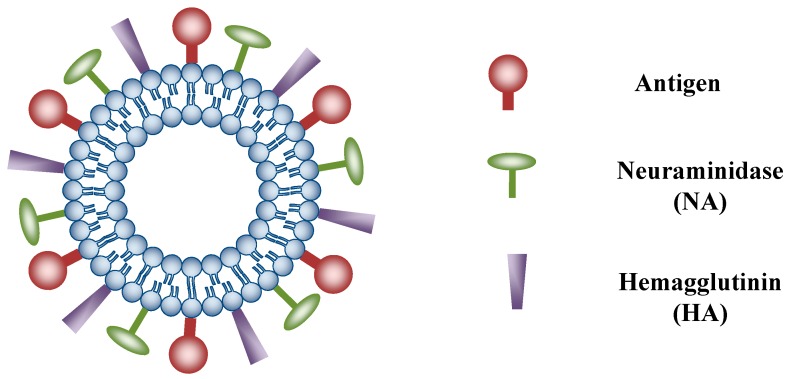
Structure of virosome. The surface of a liposome is decorated with viral surface proteins such as neuraminidase (NA), hemagglutinin (HA) and other antigens.

Currently, there are at least two approved liposomal vaccine formulations on the market for antigen delivery, including Inflexal^®^ V (influenza vaccine) and Epaxal^®^ (hepatitis A vaccine). Both formulations employed the virosomes technology (Figure 4), in which the viral proteins are bound to the surface of a liposome in an array, similar to what is seen on viral particles [171]. The idea is to create a safe viral-like particle that can induce strong protective immune response. Similar technology could be applied to DNA vaccines to improve immunogenicity. In fact, liposomes on their own could elicit immune response even in the absence of other antigens. It has been demonstrated by Lay *et al.* that DOTIM (octadecenoyloxy-ethyl-heptadecenyl-3-hydroxyethyl imidazolinium chloride)/cholesterol cationic liposome-DNA complexes (JVRS-100), in which an empty plasmid DNA vector was incorporated, were able to induce high levels of antibody and T cell immunity in mice and non-human primates [174]. Since lipid composition, particle size, surface charge and DNA entrapment efficiency of the liposomes can affect their immunogenicity and potency, these parameters must be carefully characterized and controlled. The major limitation of liposome as DNA vaccine carrier is long-term stability, as lyophilization of liposomes may not be always possible [171].

A number of studies described the use of liposomes as DNA vaccine carrier for intranasal administration. D’Souza *et al.* reported the use of a cationic and neutral co-lipid formulation, GAP-DLRIE:DOPE (aminopropyl-dimethyl-bis-dodecyloxy-propanaminium bromide-dioleoylphosphatidyl-ethanolmine) as a carrier of DNA vaccine against TB for intranasal administration [175]. Plasmid DNA encoding Ag85A was complexed with the lipids. Following intranasal immunization in mice, naked DNA was completely ineffective, probably due to the degradation of the DNA by mucosal nuclease. The lipid-DNA formulation was capable of inducing a weak cell-mediated immune response, and no humoral immune response was detected. The co-lipid intranasal formulation was compared with another cationic lipid formulation, Vaxfectin, which was used for intramuscular administration. It was found that the intramuscular formulation was able to induce a better immune response. However, combining intranasal and intramuscular administrations resulted in stronger immune responses in the lungs. Considerable improvement is needed to further develop the formulation for intranasal use.

Rosada *et al.* developed another liposome-based formulation of DNA vaccines against TB [176]. The non-toxic, cationic liposome, EPC/DOPE/DOTAP (Egg phosphatidylcholine/1,2-dioleoyl-*sn*-glycero-3-phosphoethanolamine/1,2-dioleoyl-3-trimethylammonium-propane) was used. Plasmid DNA encoding the 65 kDa mycobacterial hsp65 was either entrapped inside or complexed with the cationic liposomes, and the intramuscular and intranasal routes of administration were compared in mice. When administered intramuscularly, both liposomal formulations were ineffective in preventing tuberculosis infection in mice even after two doses. On the contrary, the complexed liposomal formulation of DNA vaccine was able to offer protection against infection even after a single dose, with a significant reduction of colony forming unit (CFU) in lungs after the immunized mice were challenged with *Mycobacterium tuberculosis*. However, four doses of intranasal administration of naked DNA vaccines failed to offer any protection. The authors reasoned that the intranasal vaccination enhance the immune response by stimulating the mucosal immunity, but the naked DNA failed to cross the mucosal barriers in the nasal cavity, demonstrating the importance with the use of delivery carrier for intranasal DNA vaccination.

Apart from TB vaccine, there are studies that reported the use of liposome to deliver influenza DNA vaccine [177,178]. Cationic liposomes DODAC/DOPE/PEG (1,2-dioleoyl-sn-glycero-3-phosphoethanolmine/dioleylphosphatidylethanolamine/polyethylene glycol) were used to encapsulate plasmid DNA encoding influenza virus HA. After intranasal immunization in mice, the liposome system was effective at eliciting both IgG and IgA humoral responses systemically. Local IgA level was enhanced. Cell-mediated immune response was also successfully induced. In addition, the immunized mice were able to withstand a lethal challenge of influenza virus. On the other hand, intramuscular immunization of the same system enhanced IgG level only with no effect on IgA level either locally or systemically. Intranasal administration of naked DNA failed to induce antibody response. The promising results demonstrated the potential of the intranasal liposomal DNA vaccine system.

To improve the DNA vaccine delivery efficiency of liposomes for intranasal administration, Khatri *et al.* modified the surface of liposomes by coating with glycol chitosan [173]. The major function of glycol chitosan is to provide mucoadhesive and immune stimulating property [179]. In the study, cationic liposomes, PC/DOPE/Chol (Phosphatidylcholine/dioleylphosphatidylethanolamine/cholesterol) were used to entrap plasmid encoding the S region of hepatitis B antigen, and the glycol chitosan was adsorbed on the liposome surfaces through electrostatic interaction and hydrogen bonding. Following intranasal administration in mice, the surface modified liposomes could elicit both humoral mucosal and cell-mediated immune responses which were better than the uncoated liposomes. Such system has the potential to be exploited for intranasal DNA vaccine of respiratory infectious diseases.

A number of studies have already demonstrated the potential of liposomal DNA vaccine system for intranasal administration, and some could offer considerable immune protection against respiratory infections in animals. However, the lipid composition of different liposomal systems varied greatly, and currently there is a lack of knowledge of how the composition may affect the immune response. To enable the utilization of liposomal DNA vaccine for clinical application and approval, a better understanding of how these factors govern the efficacy and immunity of the liposomal delivery system must first be sought.

### 5.4. Mucosal Adjuvants

To further enhance the immune responses of the DNA vaccines, adjuvants are included in the formulation in many studies. Adjuvants are generally defined as agents that could enhance the immune response of the vaccinated subjects to an antigen. In DNA vaccine, since the delivery of DNA is a major hurdle, DNA carrier system using bacterial, viral or non-viral vectors, as well as the cell specific targeting ligands, which are discussed above, are also considered as DNA vaccine adjuvants. The summary of DNA vaccine adjuvants being investigated are shown in Table 2. In this section, proteins and other macromolecules with immunopotentiating properties but are not directly involved in the delivery of DNA are discussed, especially those that are commonly employed for intranasal vaccination.

Enterotoxins are protein exotoxin released by pathogens that infect the gut. Enterotoxin-based mucosal adjuvants are the most potent and well-established strategy for the induction of both mucosal and systemic immunity to co-administered protein antigens [180]. Heat-labile enterotoxin (LT) from *E. coli* and cholera enterotoxin (CT) are very potent adjuvants but they are too toxic to be used in human. Therefore, detoxified mutants of enterotoxins have been produced by site-directed mutagenesis and they are extensively investigated as adjuvants for mucosal vaccine including intranasal vaccine. Intranasal antigen immunization with LT mutant adjuvants can provide effective protection against infectious diseases in animals [181,182,183,184,185]. It is suggested that the LT mutant adjuvants could induce potent cytotoxic T lymphocyte responses. The mechanism of action is believed to arise from enhanced permeation of antigens across epithelial barriers and a marked increase in antigen presentation by APCs [66]. Mutants of CT have also showed strong adjuvant activity [73,186,187], and could retain good adjuvant function when administered intranasally [73]. It is expected that LT mutants and CT mutants have similar mechanisms of adjuvant activities [58]. The major concern with the intranasal administration of mutant LT or CT is that these toxin derivatives may gain access into the central nervous system through the olfactory nerve. It has been reported that both native and mutant LT used as adjuvants were associated with the development of Bell’s palsy following intranasal delivery in humans [70,71,188]. The risk of enterotoxin as mucosal adjuvant for intranasal administration is already discussed in Section 3.4.

**Table 2:** Summary of mucosal adjuvants for DNA vaccine.

Types	Examples	Proposed Target or Mechanisms of Action	Reference
Enterotoxins and toxin-based derivatives	Mutants of heat-labile enterotoxin and cholera toxin	Increase antigen presentation by APCs	[187,189,190]
LPS derivatives	MLA	TLR4	[191]
Cytokines and chemokines	IL-2, IL-6, IL-7, IL-12, IL-15, GM-CSF, MCP-1, MIP-1α, RANTES	T cells stimulation Recruit and activate APCs	[192,193,194,195,196,197,198,199,200,201,202,203,204,205,206]
Oligonucleotides	CpG motifs	TLR9	[21]
Targeting ligands	Flt3 ligand DEC-205 antibody protein sigma-1	APC targeting DC targeting M cell targeting	[65,120,122,129,207,208]
Polymers	PEI	Improve DNA delivery	[146,147,148,209]
	PLGA	Improve DNA delivery	[132,133,134,166,167,210]
	Chitosan	Improve DNA delivery, mucoadhesion and immunostimulating effect	[110,155,157,158,163,211]
Liposomes	DOPE/DOTAP/PC; DOPE/PC/Chol	Improve DNA delivery plus immunostimulating effect	[175,176,212,213,214,215]

Abbreviations: LPS, lipopolysaccharide; MLA, monophosphoryl lipid A; GM-CSF, granulocyte-macrophage colony-stimulating factor; MCP-1, monocyte chemoattractant protein-1; MIP-1α, macrophage inflammatory protein-1α; APCs, antigen presenting cells.

Lipopolysaccharides (LPS) are the major component of the outer membrane of Gram-negative bacteria, and could elicit strong immune response. However, they are also highly toxic. In order to make them safe and suitable as vaccine adjuvants, LPS derivatives are produced to reduce the endotoxic effect but to retain their immunostimulatory function. Monophosphoryl lipid A (MPL) is one example of LPS derivative that is investigated as vaccine adjuvant. MPL is prepared by removal of a phosphate and fatty acid group from the lipid A of *Salmonella minnesola*. MPL is thought to interact with Toll-like receptor 4 (TLR-4) on APCs. It has been demonstrated that MPL could activate macrophages, increase their cytokine secretion and hence immune response [216,217,218,219,220]. Regarding safety, MPL appears to retain the immunogenic activity of LPS but with significantly reduced toxicity [221]. MPL has been extensively evaluated in the clinic as adjuvants for various diseases including infectious diseases with an acceptable profile of adverse effects [182]. MPL has been used successfully as a mucosal adjuvant when formulated with liposomes or virosomes for intranasal administration in animals [222,223,224].

Cytokines are small proteins that are important in regulating immunological response by recruiting and stimulating T cells, or by directly acting on infected cells. They have potential to be natural adjuvants for DNA vaccines [225]. Cytokines that have been evaluated as possible DNA or intranasal vaccine adjuvants include IL-1, IL-2, IL-6, IL-12, IL-15 and GM-CSF [205,206,226,227]. In particular, IL-12 is found to be an effective mucosal adjuvant [228,229]. Lynch *et al.* demonstrated that intranasal administration of pneumococcal polysaccharide conjugate vaccine in the presence of IL-12 was able to enhance systemic and mucosal immune responses to pneumococci in mice [228]. However, cytokines have short half-life *in vivo* and poor stability. They are also expensive and are associated with dose related toxicity. Therefore, these molecules may not be suitable for use as adjuvants in vaccines designed to protect against infectious diseases [182]. Nevertheless, intranasal administration of IL-12 induced less toxicity than parenteral administration [230]. Alternatively, cytokines can be expressed from plasmid DNA to allow long-lasting expression *in vivo* and to reduce the cost of production [196,198].

## 6. Concluding Remarks

With the emergence of new or antimicrobial-resistant bacteria and viruses, and the ease of transmission, especially the respiratory pathogens, respiratory infections are becoming serious threats to human health. Safe and effective vaccines are important to safeguard public health. Intranasal DNA vaccination appears to be a promising non-invasive approach to provide protection against various infectious diseases. Evidence shows that intranasal DNA vaccine could elicit strong and long-lasting humoral as well as cell-mediated immune responses in many animal models. DNA vaccines are already successfully used in veterinary products for protection against infections, but their immunogenicity needs to be further enhanced to make them suitable for human use. Improving DNA delivery and formulation is one of the several strategies to enhance the immune response. Various studies have demonstrated that significant improvement of immune response that could be achieved by the employment of DNA carrier system, or to target the DNA vaccines to APCs. DNA vaccines generally have good safety profile, but the potential toxicity associated with DNA delivery systems, especially when they are used at high concentration, must not be neglected. DNA vaccines may circumvent many problems associated with conventional vaccines such as high costs of protein vaccine purification and bacterial/viral inactivated or attenuated process, the incorrect folding of antigen and viral mutation risk, thereby offering a safer alternative to benefit humans. In addition, mass manufacture of DNA vaccine is easier and faster, and DNA product is usually highly stable. Once an effective intranasal DNA vaccine delivery system is identified and optimized, a delivery technology platform could be established to allow the development of DNA vaccine formulations for different infectious diseases in the future.
